# Fire Simulation and Cardiovascular Health in Firefighters

**DOI:** 10.1161/CIRCULATIONAHA.116.025711

**Published:** 2017-04-03

**Authors:** Amanda L. Hunter, Anoop S.V. Shah, Jeremy P. Langrish, Jennifer B. Raftis, Andrew J. Lucking, Mairi Brittan, Sowmya Venkatasubramanian, Catherine L. Stables, Dominik Stelzle, James Marshall, Richard Graveling, Andrew D. Flapan, David E. Newby, Nicholas L. Mills

**Affiliations:** From British Heart Foundation Centre for Cardiovascular Science, University of Edinburgh, United Kingdom (A.L.H., A.S.V.S., J.P.L., A.J.L., M.B., S.V., C.L.S., D.S., D.E.N., N.L.M.); ELEGI/Colt Laboratories, Medical Research Council/University of Edinburgh Centre for Inflammation Research, Queens Medical Research Institute, United Kingdom (J.B.R.); Scottish Fire and Rescue Service, Edinburgh, United Kingdom (J.M.); Institute of Occupational Medicine, Edinburgh, United Kingdom (R.G.); and Edinburgh Heart Centre, Royal Infirmary of Edinburgh, United Kingdom (A.D.F.).

**Keywords:** firefighters, thrombosis, vascular, endothelium-dependent relaxation

## Abstract

**Background:**

Rates of myocardial infarction in firefighters are increased during fire suppression duties, and are likely to reflect a combination of factors including extreme physical exertion and heat exposure. We assessed the effects of simulated fire suppression on measures of cardiovascular health in healthy firefighters.

**Methods:**

In an open-label randomized crossover study, 19 healthy firefighters (age, 41±7 years; 16 males) performed a standardized training exercise in a fire simulation facility or light duties for 20 minutes. After each exposure, ex vivo thrombus formation, fibrinolysis, platelet activation, and forearm blood flow in response to intra-arterial infusions of endothelial-dependent and -independent vasodilators were measured.

**Results:**

After fire simulation training, core temperature increased (1.0±0.1°C) and weight reduced (0.46±0.14 kg, *P*<0.001 for both). In comparison with control, exposure to fire simulation increased thrombus formation under low-shear (73±14%) and high-shear (66±14%) conditions (*P*<0.001 for both) and increased platelet-monocyte binding (7±10%, *P*=0.03). There was a dose-dependent increase in forearm blood flow with all vasodilators (*P*<0.001), which was attenuated by fire simulation in response to acetylcholine (*P*=0.01) and sodium nitroprusside (*P*=0.004). This was associated with a rise in fibrinolytic capacity, asymptomatic myocardial ischemia, and an increase in plasma cardiac troponin I concentrations (1.4 [0.8–2.5] versus 3.0 [1.7–6.4] ng/L, *P*=0.010).

**Conclusions:**

Exposure to extreme heat and physical exertion during fire suppression activates platelets, increases thrombus formation, impairs vascular function, and promotes myocardial ischemia and injury in healthy firefighters. Our findings provide pathogenic mechanisms to explain the association between fire suppression activity and acute myocardial infarction in firefighters.

**Clinical Trial Registration:**

URL: http://www.clinicaltrials.gov. Unique identifier: NCT01812317.

**Editorial, see p 1296**

Cardiovascular events are the leading cause of death among firefighters and are responsible for ≈45% of on-duty fatalities each year in the United States.^1^ These deaths disproportionately cluster around fire suppression duties, despite this activity accounting for only 1% to 5% of a firefighter’s time.^2^ Death from coronary artery disease was 12 to 136 times more likely to occur during or shortly after fire suppression than nonemergency duties. The hostile conditions of fire suppression include high ambient temperatures, extreme physical exertion, noxious air pollutants, and psychological stress. It is likely that there are additive or synergistic effects of these potential triggers in susceptible firefighters that may culminate in acute cardiovascular events.

Fire training forms an important part of the recruitment process and training among operational firefighters. The cardiovascular response to fire suppression is difficult to study in a real-life setting because of the unpredictability of events and time pressures during an emergency situation. As a research tool, real fire training centers offer a unique opportunity to assess the physiological effects of fire suppression in a controlled setting.^3–15^ Previous observations suggest that heat and physical stress, in combination with hemoconcentration attributable to fluid loss, reduce cardiac output and induce a hypercoagulable state.^3,16^ However, our understanding of the pathogenic mechanisms to explain the association between fire suppression and acute myocardial infarction is incomplete, and, therefore, the optimal approach to reduce risk in firefighters is uncertain. Our aim was to undertake a comprehensive assessment of the effects of fire suppression on cardiovascular health in firefighters.

## Methods

### Study Participants

Nineteen healthy nonsmoking firefighters were enrolled in the study. The study was performed in accordance with the Declaration of Helsinki, with the approval of the local research ethics committee and the written informed consent of all volunteers. Subjects were recruited by sending study information sheets and letters to randomly selected firefighters from the Scottish Fire and Rescue Service. Exclusion criteria included cigarette smoking, known cardiovascular disease, arrhythmias, diabetes mellitus, hypertension, asthma, use of regular medication, renal or hepatic impairment, or an intercurrent infective illness. Subjects reported no symptoms of respiratory tract infection within the 4-week period preceding the study.

### Study Design

Subjects attended on 2 occasions, at least 1 week apart, and participated in a standardized training exercise in a fire simulation facility (exposure) or undertook light duties (control) in an open-label, randomized crossover design. Firefighters attended after a period of 48 hours off-duty to minimize the impact of confounding from other occupational exposures. Fire simulation exposure was performed at a separate dedicated training facility before transportation to the clinical research facility. During the control period, firefighters attended the clinical research facility and were permitted to undertake light activity similar to that performed during a shift without emergency duties.

After each exposure, cardiovascular assessments were performed in a quiet, temperature-controlled room maintained at 22°C to 24°C with subjects lying supine. All subjects abstained from alcohol for 24 hours and from food, tobacco, and caffeine-containing drinks for at least 4 hours before each vascular study. Female subjects were assessed at the same time point of their menstrual cycle.

The primary end points were ex vivo thrombus formation, forearm blood flow, and net t-PA (tissue-type plasminogen activator) release. Secondary end points were platelet activation assessed by flow cytometry, differential cell count, plasma urea, creatinine, lactate, glucose, and cardiac troponin I concentrations, ambulatory heart rate and blood pressure monitoring, and ischemic burden on 12-lead Holter monitoring.

On the basis of previous studies,^17–20^ ex vivo thrombus formation was assessed 1 to 2 hours after each exposure, and forearm blood flow and t-PA release were assessed 2 to 4 hours after each exposure. Blood samples were obtained at baseline, immediately, 4 hours, and 24 hours postexposure.

Subjects were fitted with a portable 12-lead ECG (Lifecard CF, Delmar Reynolds Medical Ltd) and blood pressure monitor (Spacelabs 90217, Spacelabs) at least 30 minutes before and for 24 hours after exposure. On the evening before the fire simulation training, firefighters were asked to swallow an ingestible temperature monitor (CorTemp, HQInc). In cases where the pill was no longer in their gastrointestinal tract when arriving at the study site, subjects swallowed a second pill ≈30 minutes before study commencement. Core body temperature was measured continuously with externally worn temperature loggers (CorTemp, HQInc) from 30 minutes before exposure, throughout the exposure, and for at least 6 hours thereafter. All subjects wore full personal protective equipment and self-contained breathing apparatus for the fire simulation exposure.

Overall sweat loss was determined by the difference in body mass between the beginning and end of the exposure, and was corrected for any fluid consumption. Firefighters voided urine before being weighed before the fire simulation exposure. Participants reported their ratings of perceived exertion, from 6 to 20 (very light to very, very difficult) on the Borg scale immediately postexposure.^21^

### Exposures

Firefighters attended the Scottish International Fire Training Center, Edinburgh, for the fire simulation exposure. This is a specially designed facility consisting of steel shipping containers bolted together on 2 levels with the internal layout of a dwelling house. Fires are ignited simultaneously in 1 or 2 rooms of the facility 15 minutes before the fire simulation exercise starts. Internal temperature is monitored throughout the facility at 0.5, 1.0, and 1.5 m above floor level. Fire simulation exposure was undertaken as a standardized exercise for a median duration of 20 minutes 22 s (range, 19 minutes 42 s to 21 minutes 6 s). All participants undertook the same tasks in the same order. This comprised entering the facility as part of a team of 4 firefighters, ascending stairs while dragging a water-filled hose throughout the facility, locating and attempting to extinguish the fire located on the first floor, before identifying and rescuing a casualty. Casualties took the form of 80-kg dummies on the ground floor that were lifted with assistance from a second firefighter and removed from the facility. The exposure was determined to be complete by the supervising instructor as soon as the firefighter stepped outside the exposure facility. The firefighter was immediately escorted to an adjacent outbuilding where postexposure assessments were undertaken. After the exposure, firefighters removed their personal protective equipment and self-contained breathing apparatus. They were advised to rehydrate as they normally would after this exercise and to measure the volume of fluid they ingested. Weight after the exposure was corrected for the volume of fluid ingested to determine losses during fire simulation.

### Ex Vivo Thrombosis Studies

Thrombus formation was measured using the Badimon chamber as described previously.^18,19^ In brief, a pump was used to draw blood from an antecubital vein through a series of 3 cylindrical perfusion chambers maintained at 37°C in a water bath. Carefully prepared strips of porcine aorta, from which the intima and a thin layer of media had been removed, acted as the thrombogenic substrate. Each study lasted for 5 minutes during which flow was maintained at a constant rate of 10 mL/min. Porcine strips with thrombus attached were removed and fixed in 4% paraformaldehyde. Strips were wax embedded, sectioned, and stained with Masson Trichrome. Images were acquired at ×20 magnification, and the thrombus area was measured using an Ariol image acquisition system (Leica Microsystems GmbH) by a blinded operator. Results from at least 6 sections were averaged to determine thrombus area for each chamber, as described previously.^18,19^

### Flow Cytometry

Blood was taken from an antecubital vein using a 21-gauge cannula and anticoagulated with d-phenylalanyl-l-prolyl-l-arginine chloromethylketone (75 μm; Cambridge Biosciences) as previously described.^22^ Samples were not analyzed unless venesection achieved rapid and uninterrupted blood flow. Five minutes after sample collection, samples were stained with the following conjugated monoclonal antibodies: allophycocyanin-conjugated CD14, allophycocyanin-conjugated CD36, phycoerythrin-conjugated CD62P, and phycoerythrin-conjugated CD154; phycoerythrin-conjugated CD40, fluorescein isothiocyanate–conjugated CD42a, and appropriate control isotypes (all Becton Dickinson). All antibodies were diluted 1:20. Once stained, samples were incubated for 20 minutes at room temperature to identify P-selectin and CD40L on the platelet surface and CD40 on the monocyte surface. Platelet-monocyte samples were fixed with FACS-Lyse (Becton Dickinson). Platelet samples were fixed with 1% paraformaldehyde. Samples were analyzed within 24 hours using a FACSCalibur flow cytometer (Becton Dickinson). Platelet-monocyte aggregates were defined as monocytes positive for CD14. Data analysis was performed using FlowJo (Treestar).

### Vasomotor and Fibrinolytic Studies

All subjects underwent brachial artery cannulation with a 27-standard wire gauge steel needle under controlled conditions. After a 30-minute baseline saline infusion, acetylcholine at 5, 10, and 20 μg/min (endothelium-dependent vasodilator that does not release t-PA; Merck Biosciences); bradykinin at 100, 300, and 1000 pmol/min (endothelium-dependent vasodilator that releases t-PA; American Peptide Company); sodium nitroprusside at 2, 4, and 8 μg/min (endothelium-independent vasodilator that does not release t-PA; Hospira, Inc); and verapamil at 10, 30, and 100 μg/min (endothelium- and nitric oxide–independent vasodilator that does not release t-PA; BGP Products Ltd) were infused for 6 minutes at each dose. The 4 vasodilators were separated by 20-minute saline infusions and given in a randomized order, with the exception of verapamil, which was always given last because of the longer duration of action.^23^ Forearm blood flow was measured in infused and noninfused arms by venous occlusion plethysmography with mercury-in-silicone elastomer strain gauges as described previously.^24^

Venous cannulas (17 gauge) were inserted into large subcutaneous veins of the antecubital fossae of both arms. Blood (10 mL) was withdrawn simultaneously from each arm at baseline and during infusion of each dose of bradykinin and collected into acidified buffered citrate (Stabilyte tubes, Biopool International) for t-PA assays and into citrate (BD Vacutainer) for plasminogen activator inhibitor type 1 assays. Samples were kept on ice before being centrifuged at 2000*g* for 30 minutes at 4°C. Platelet-free plasma was decanted and stored at –80°C before assay.

Plasma t-PA and plasminogen activator inhibitor type 1 antigen concentrations were determined by enzyme-linked immunosorbant assays (TECHNOZYM t-PA Combi Actibind, Technoclone; Zymutest plasminogen activator inhibitor type 1, Hyphen BioMed). Hematocrit was determined by capillary tube centrifugation at baseline and during infusion of bradykinin at 1000 pmol/min.

### Assays

Venous blood was analyzed for total cells, differential cell counts, and platelets by an autoanalyzer and for plasma urea and electrolytes, lactate, and glucose concentration in the regional laboratories at the Royal Infirmary of Edinburgh. Plasma cardiac troponin I concentrations were determined using a high-sensitivity assay (ARCHITECT_*STAT*_, Abbott Diagnostics). This assay has a limit of detection of 1.2 ng/L, an upper reference limit (99th centile) of 34 ng/L in men and 16 ng/L in women.^25^ We previously reported an interassay coefficient of variation of 12.6% at 3.5 ng/L.^26^

### ECG Analysis

Electrographic recordings were analyzed with the use of the Medical Pathfinder Digital 700 Series Analysis System (Delmar Reynolds Medical Ltd) as previously described.^27^ A single operator, blinded to both subject characteristics and exposure, verified any abnormal rhythms and manually edited artifact and aberrant beats. Holter monitors were attached at least 30 minutes before the exposure period. This 30-minute period was used to normalize the ST segment for each lead during a rest period. Thereafter, ST-segment deviation was calculated by comparing the ST-segment amplitude during the first 60 minutes (including the 20-minute exposure period) and then across the subsequent 23-hour period. The ST-segment amplitude was determined at the J-point plus 50 ms; 0.5 mm events were defined as any episode with ≥0.5 mm ST-segment depression lasting at least 1 minute. The ischemic burden during each exposure was calculated as the product of the change in the ST-segment amplitude and the duration of the exposure. Leads II, V_2_, and V_5_ were selected a priori for ST-segment analysis to reflect separate regions of the myocardium. The maximum ST-segment depression and ischemic burden were determined for each lead and as a composite.^27^

### Data Analysis and Statistics

Continuous variables are reported as mean±standard error of the mean. Statistical analyses were performed with GraphPad Prism, version 5.0 (Graph Pad Software) by 2-way analysis of variance with repeated measures and 2-tailed Student paired *t* test, or Wilcoxon signed-rank as appropriate. Cardiac troponin I concentrations were log transformed before analysis. Statistical significance was taken at 2-sided *P*<0.05.

## Results

Nineteen healthy nonsmoking firefighters (mean age, 41±7 years; 16 males) were enrolled (Table 1). Seventeen firefighters had both exposures; 2 firefighters unable to complete the study were excluded.

**Table 1.**
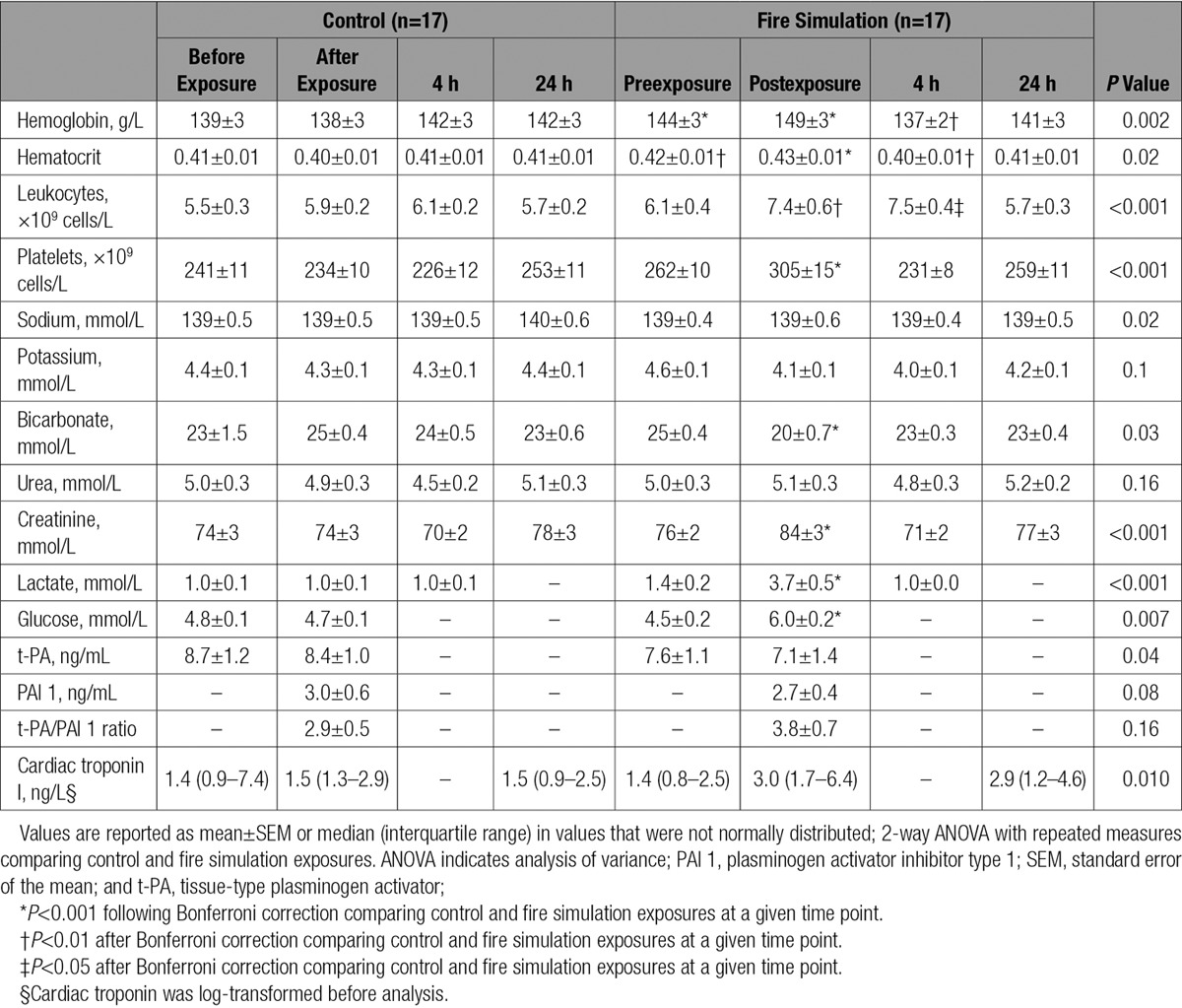
Secondary End Points Before and After Control and Fire Simulation Exposures

**Figure 1.**
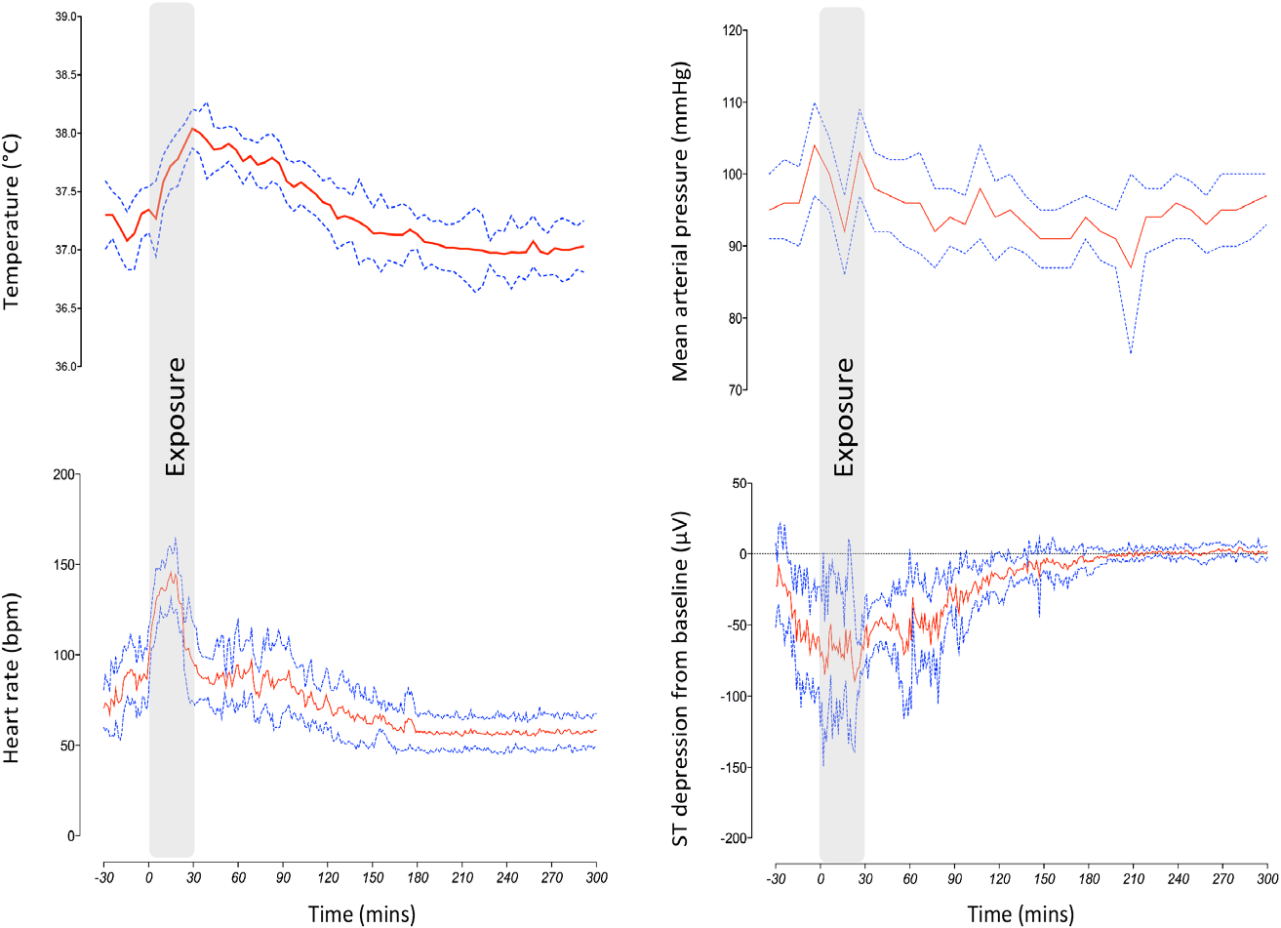
**Core temperature and hemodynamic changes during fire simulation exposure.** Core temperature and heart rate rapidly increased during the fire simulation accompanied by asymptomatic ST-segment depression. Core temperature remained increased for 120 minutes, and ST segments returned to baseline gradually during the same time period. Mean arterial pressure was unchanged throughout. Values are mean±95% confidence interval.

The average maximum temperature in the fire simulation facility was 406.2±13.5°C. Mean core temperature was at 37.4±0.1°C at baseline and peaked at 38.4±0.1°C. Body weight reduced by 0.46±0.14 kg. Heart rate and temperature rose rapidly and were associated with asymptomatic ST-segment depression during fire simulation exposure (analysis of variance *P*<0.01 for all; Figure 1). Perceived exertion was rated as 14±0.2 on the Borg scale.

### Thrombus Formation and Platelet Activation

**Figure 2.**
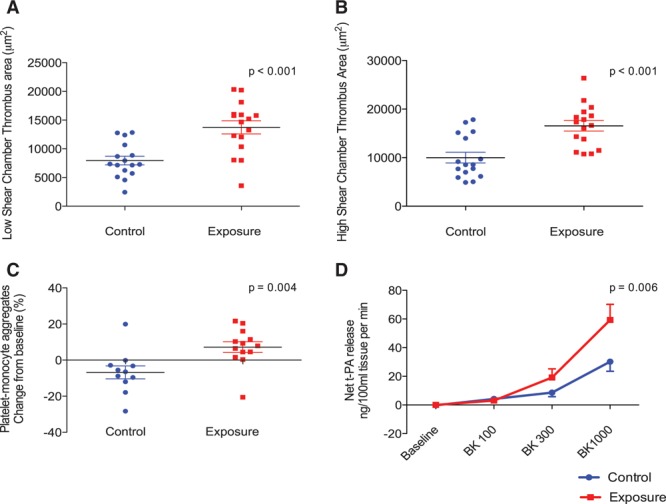
**Thrombus formation and platelet activation after fire simulation exposure.** Thrombus formation ex vivo was increased in response to fire simulation in under both low-shear and high-shear conditions in comparison with control (Student *t* test, *P*<0.001 for both, **A** and **B**, respectively). Platelet-monocyte aggregates were increased after fire simulation exposure in comparison with control (Student *t* test, *P*=0.004, n=12, **C**). Where venipuncture and sample processing resulted in significant ex vivo platelet activation, data were excluded from further analysis by a researcher blinded to the exposure. There was a dose-dependent increase in t-PA (tissue-type plasminogen activator) in response to bradykinin in both exposures (2-way ANOVA with repeated measures, *P*<0.001), that was augmented after fire simulation in comparison with control (ANOVA, *P*=0.006, **D**). ANOVA indicates analysis of variance.

In comparison with the control period, thrombus formation was increased after fire simulation exposure by 73% in the low-shear chamber (change in thrombus area 5781 μm^2^; 95% confidence interval [CI], 3340–8221 μm^2^; *P*<0.001) and by 66% in the high-shear chamber (change in thrombus area 6563 μm^2^; 95% CI, 3481–9645 μm^2^; *P*<0.001, Figure 2). Platelet-monocyte aggregation differed at baseline between control and fire simulation exposure, but was increased after fire simulation (7%; 95% CI, 0%–13%; *P*=0.03) and was unchanged across the control period (–6%; 95% CI, –15% to 1%; *P*=0.09, Figure 2). Platelet surface expression of P-selectin and CD40 ligand were similar after control and fire simulation exposure (*P*>0.05 for both).

### Vascular Vasomotor and Fibrinolytic Function

**Figure 3.**
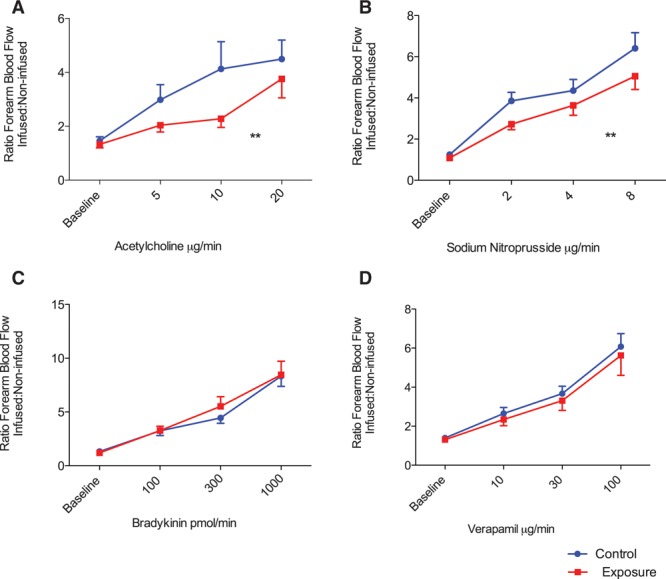
**Vascular vasomotor function after fire simulation exposure.** There was a dose-dependent increase in forearm blood flow with each vasodilator (2-way ANOVA with repeated measures, *P*<0.001 for all). Vasodilatation expressed as a ratio of the forearm blood flow between the infused and noninfused arm, was attenuated in response to acetylcholine and sodium nitroprusside (*P*=0.01 and *P*=0.004, **A** and **B**, respectively) after fire simulation in comparison with control. There was no difference in forearm blood flow in response to bradykinin or verapamil (*P*>0.05 for both, **C** and **D**, respectively) between the 2 exposures. **P<0.01. ANOVA indicates analysis of variance.

After fire simulation exposure, both systolic and diastolic blood pressures were lower immediately before the vascular studies in comparison with the control period (systolic blood pressure 125±2 versus 134±3 mm Hg, diastolic blood pressure 75±2 versus 82±2 mm Hg; *P*<0.01 for both). Basal forearm blood flow was higher at baseline after fire simulation exposure in comparison with the control period (2.3±0.2 versus 1.7±0.1 mL·100 mL^–1^·min^–1^; *P*=0.01). After the administration of acetylcholine, bradykinin, sodium nitroprusside, and verapamil, there were dose-dependent increases in forearm blood flow after both fire simulation and control periods (*P*<0.001, Figure 3). Vasodilatation expressed as a ratio of forearm blood flow in the infused and control arms was attenuated in response to acetylcholine (*P*=0.01) and sodium nitroprusside (*P*=0.004) in comparison with control, but was unaffected by bradykinin or verapamil infusions (*P*>0.05 for both).

Bradykinin caused a dose-dependent increase in plasma t-PA antigen concentrations (*P*<0.001). After fire simulation exposure, there was a doubling of the net release of t-PA antigen in comparison with control (area under the curve, 51.9 versus 27.9 ng·100 mL^–1^·min^–1^; *P*=0.006, Figure 2).

### Secondary End Points

There were increases in hemoglobin, hematocrit, platelets, and total leukocytes immediately after fire simulation in comparison with control (hemoglobin 149±3 versus 138±3 g/L, hematocrit 0.43±0.01 versus 0.40±0.01, platelets 305±15 versus 234±10×10^9^ cells/L, total leukocytes 7.4±0.6 versus 5.9±0.2×10^9^ cells/L; *P*<0.05 for all, Table 1). Total leukocytes remained elevated at 6 hours postexposure in comparison with the control period (7.5±0.4 versus 6.1±0.2×10^9^ cells/L; *P*<0.05). Serum lactate was increased immediately after fire simulation exposure reflected by a corresponding fall in bicarbonate concentrations (lactate 3.7±0.5 versus 1.0±0.1 mmol/L, bicarbonate 20±0.7 versus 25±0.4 mmol/L; *P*<0.05 for both).

High-sensitivity cardiac troponin I concentration increased 1 hour after fire simulation in comparison with control (3.0 [1.7–6.4] versus 1.5 [1.3–2.9])ng/L, *P*=0.010). There was a significant increase in the number of episodes of ST-segment depression exceeding 0.5 mm, maximum ST-segment depression, and cumulative ischemic burden during fire simulation exposure in comparison with the control period (*P*<0.05 for all), but these parameters did not differ across the subsequent 23-hour period (Table 2).

**Table 2.**
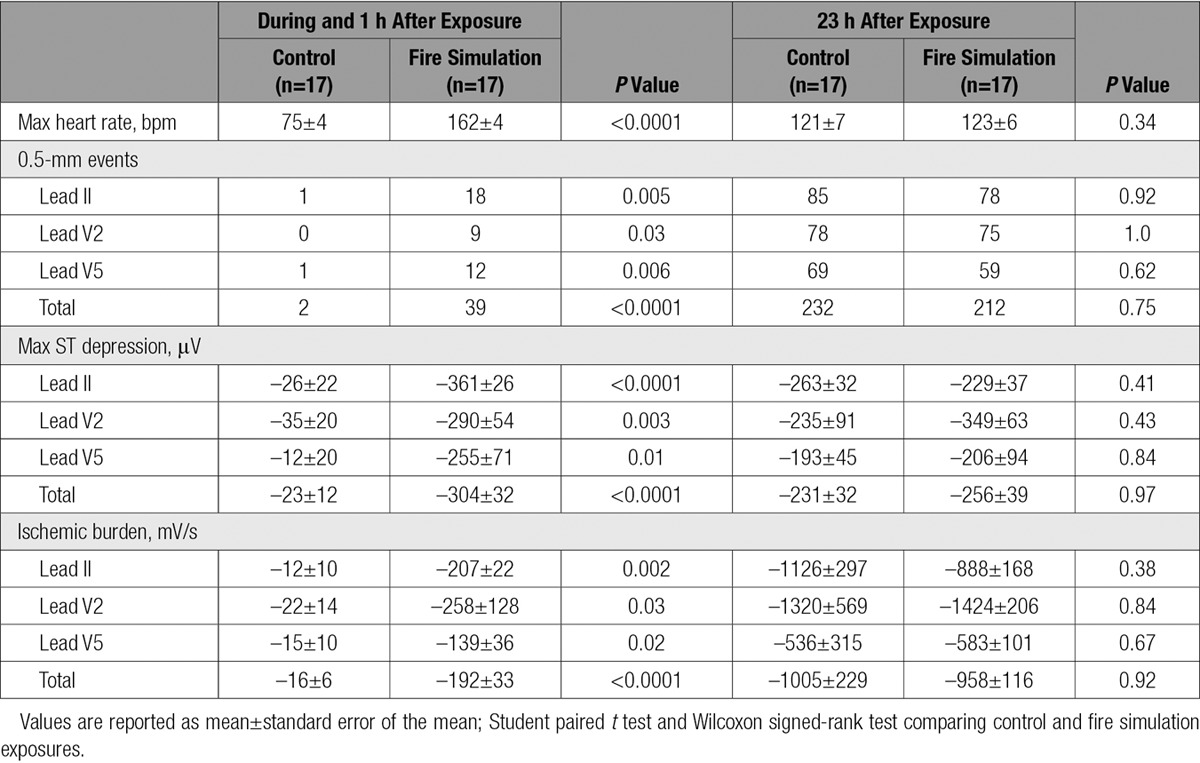
Continuous 12-Lead Electrographic Analysis

## Discussion

After a short exposure to fire simulation training, we demonstrate changes in physiological measures of cardiovascular function. This is the first study to demonstrate that participation in fire simulation results in increased ex vivo thrombus formation along with enhanced platelet activation and impairment of vasomotor endothelial function: all key mechanisms in the pathogenesis of acute myocardial infarction. It is intriguing that fire simulation was associated with evidence of minor myocardial injury and asymptomatic myocardial ischemia. Taken together, this comprehensive cardiovascular assessment has detected plausible mechanistic links between participation in fire suppression duties and acute myocardial infarction.

Participation in fire simulation training places an inordinate strain on the cardiovascular system. By undertaking a comprehensive physiological assessment that included continuous core body temperature and hemodynamic monitoring, we are able to demonstrate the effect that heat and physical exertion has on a range of physiological and cardiovascular health measures. We reveal a significant loss of body weight secondary to dehydration in association with a temperature rise of 1°C, lactic acidosis, and leukocytosis. It is unsurprising that this was associated with an acute increase in heart rate and relative hypotension over the following 3 to 4 hours as the subjects returned to normothermia.

For the first time, we demonstrate an increase in ex vivo thrombus formation in response to fire simulation exposure that is perhaps not surprising given the associated hemoconcentration. The Badimon chamber is a well-validated ex vivo model of thrombosis permitting measurement of thrombus formation in native whole blood in conditions that simulate coronary arteries after plaque disruption.^18,28,29^ The degree of thrombus formation demonstrated in healthy firefighters after a short fire simulation exposure is notable. Our group has previously reported an increase in ex vivo thrombus formation after diesel exhaust exposure,^18^ but the degree of thrombus formation in the current study was >3-fold greater. This is likely to be explained by systemic inflammation together with presumed sympathetic activation, the combination of which stimulates platelets and activates coagulation pathways. Moreover, the increased thrombogenicity attributable to dehydration further compounds this effect, resulting in a substantial increase in thrombus formation. Others have previously demonstrated a 24% increase in platelet count and an increase in platelet aggregability.^11^ Alongside demonstrating a 31% increase in platelet count, we use a more robust assessment of platelet activation by flow cytometry and have demonstrated an increase in platelet-monocyte aggregates after fire simulation, suggesting that increased thrombus formation is at least in part mediated by platelet activation.

We observed an increase in endothelial tissue plasminogen activator release from the vascular endothelium in response to intra-arterial bradykinin infusion after fire simulation exposure, and this is likely to be compensatory in response to the marked prothrombotic state. Previous studies have shown that plasma t-PA antigen concentrations increase in parallel with increased coagulation in the early phase after both submaximal exercise^30,31^ and a similar fire simulation exposure.^7^ However this fibrinolytic response diminishes within 2 hours with a persistent imbalance of thrombosis and fibrinolysis in favor of a prothrombotic state. This perhaps explains the ongoing susceptibility to cardiac events in firefighters beyond the immediate postexposure period. Furthermore, it has been previously established that creating a proinflammatory state in the vascular endothelium results in a sustained and substantial increase in endothelial tissue plasminogen activator while simultaneously impairing endothelial vasomotor function.^32^ A limitation of our study is that we did not assess t-PA release at a later time point, when any systemic inflammatory response to fire simulation is likely to be more marked.

Previous work by Tei and colleagues^33^ has demonstrated that exposure to heat alone in the form of a hot water bath or sauna, increased core body temperature by 1.2°C and reduced systemic vascular resistance during and for up to 30 minutes after exposure in patients with heart failure. The authors conclude that regular heat exposure could have a beneficial effect on cardiovascular physiology in these patients. Although we demonstrate a similar increase in core temperature after fire simulation, the additional physical and psychological effects of fire suppression are distinct. Furthermore, we evaluated vascular function 2 hours after exposure when core body temperature had nearly returned to baseline. In a setting similar to our study, Fahs and colleagues^5^ revealed an increase in arterial stiffness together with increases in forearm blood flow and reactive hyperemia after fire simulation as measured noninvasively by venous occlusion plethysmography. Limited conclusions were drawn from this study given the apparently opposing vascular effects demonstrated. In contrast, we used venous occlusion plethysmography with intra-arterial infusion of vasodilators, widely regarded as the gold-standard assessment of endothelial vasomotor function. Although forearm blood flow after fire simulation exposure was elevated because of systemic vasodilatation, we demonstrated a detrimental effect on endothelium-dependent and -independent vasodilatation after exposure to fire simulation by reporting the change in blood flow as a ratio of the infused to noninfused arms to account for systemic vasodilatation.

The endothelium is a major target for inflammation and consequent oxidative stress with impairment of endothelial vasomotor function being associated with an increased risk of acute cardiovascular events, including cardiovascular death.^34,35^ It is conceivable that the proinflammatory state created by exposure to fire simulation accounts for the attenuated response to acetylcholine and sodium nitroprusside whose vasodilatory actions are mediated by nitric oxide. We postulate that oxygen free radicals scavenge nitric oxide, thus reducing its bioavailability. By contrast, there was no impairment of vasomotor function with bradykinin or verapamil after either exposure. This would suggest that impaired vasodilatation to acetylcholine and sodium nitroprusside is not simply a manifestation of altered basal tone or systemic vasodilatation. Bradykinin causes vasodilatation primarily through the release of endothelium-derived hyperpolarizing factor and prostaglandins, and therefore the vasomotor response to bradykinin infusion may be less susceptible to the acute effects of oxidative stress. Alternatively, active vasodilatation during whole body heat stress may be mediated by the cyclooxygenase pathway,^36^ which could also explain the lack of attenuation in forearm vasomotor response to bradykinin after fire simulation exposure. It is likely that vasodilatation is mediated by upregulated prostanoids in this setting and counteracts the impairment of nitric oxide–mediated dilatation resulting in a neutral response to bradykinin administration.

We have demonstrated small increases in plasma high-sensitivity troponin I concentrations after fire suppression exposure. Previous studies have reported a link between endurance exercise and cardiac troponin release.^37–39^ The magnitude of increase in troponin postexercise is related to exercise intensity and cardiovascular physiology. In a recent meta-analysis, there was a pooled increase in cardiac troponin I from baseline of 40 ng/L (95% CI, 21.4–58 ng/L) and in cardiac troponin T of 26 ng/L (95% CI, 5.2–46) after prolonged endurance exercise with a mean exercise duration of 229 minutes.^39^ We used a high-sensitivity cardiac troponin I assay with excellent precision at very low concentrations in this study^26,40^ and were able to observe a small increase in cardiac troponin I concentrations in all subjects after only 20 minutes of fire suppression training. In addition, we demonstrate ST-segment depression on ambulatory monitoring and periods of asymptomatic myocardial ischemia during and immediately after fire simulation exposure. The firefighters in this study were healthy with no risk factors for, and no known underlying coronary artery disease. Although cardiac troponin I concentrations remained within the normal reference range and the degree of myocardial ischemia was relatively small, it is plausible that these changes represent direct cardiac injury and the cardiotoxic effect of cytokines such as tumor necrosis factor, heat shock protein, or oxygen free radicals. Alternatively this may represent an oxygen supply-demand mismatch causing myocardial injury at the extremes of physical exertion.^41^ Further studies would be required comparing the effects of fire suppression with the effects of an equivalent period of physical exercise in the absence of fire suppression to determine the mechanism of myocardial injury.

The measured maximal heart rates in this study were similar to those in other fire simulation studies.^10,16,42–44^ Despite the apparent strenuous exertion, the fire simulation exposure was graded by subjects as strenuous, yet not very hard on the Borg Scale. Ratings of perceived exertion are commonly used in simulated real fire exercises. However, there is generally poor correlation between perceived exertion and heart rate, with most subjects grading exercises as less strenuous than their heart rates would otherwise suggest. This raises an important safety issue and questions if firefighters are aware they are working at the limits of their physiological capabilities.

Fire simulation exposure undoubtedly is not accurately representative of real-life fire suppression, which is the main limitation of this study. In real-life fire suppression, the physiological stresses demonstrated in this fire simulation will undoubtedly be compounded by uncontrolled and higher ambient temperatures, multiple entries into the same fire, and the potential psychological stress of attending an unknown and dangerous situation where one’s life and the lives of others are at risk. All firefighters involved in this study were familiar with the fire simulation center and the exercise undertaken owing to previous attendances for annual training. If we can extrapolate the findings of this study to a real-life fire suppression scenario, we would surmise that firefighters would have higher core temperatures, given a higher ambient temperature, that are unable to return to baseline given multiple entries to the same fire within a short time frame when there is often inadequate time for active cooling or rehydration before reentry. Further studies are required outside a fire training facility to assess the effects of real-life fire suppression that will encompass the additional triggers of psychological stress and air pollution, not assessed here, but undertaking such studies will undoubtedly prove logistically challenging. In addition, although we have been able to demonstrate that the combination of extreme heat and physical exertion is detrimental to many measures of cardiovascular function, we did not undertake a comparison of fire simulation exposure to an exposure consisting of either heat or exercise alone to assess the effect of each individual component on cardiovascular function. However, in this study we wished to simulate the effects of a real-life fire suppression activity as closely as possible, and, in reality, firefighters are never exposed to heat without physical exertion and neither is avoidable for them. Further experimental studies would be required to be undertaken to explore each of these components separately.

Our study has important implications for firefighters participating in fire simulation training. If the increased thrombogenicity and impaired vascular function observed in our study is secondary to an increase in core body temperature and dehydration, then limiting the duration of exposure, active cooling, and effective rehydration would be simple and inexpensive ways to mitigate the risk posed by fire simulation training.

In conclusion, exposure to extreme heat and physical exertion during simulated fire suppression increases thrombogenicity, impairs vascular function, and causes myocardial injury in healthy firefighters. Our findings suggest the pathogenic mechanisms to explain the association between fire suppression activity and acute myocardial infarction in susceptible firefighters.

## Acknowledgments

The authors thank the nursing staff at the Clinical Research Facility at the Royal Infirmary of Edinburgh for their assistance with these studies. The authors give thanks to the staff at the Scottish Fire Training Center, Edinburgh, for their invaluable assistance in running the exposure facility. Drs Mills and Hunter conceived and participated in the design of the study. Drs Hunter, Shah, and Langrish performed the clinical studies and collected the data. Drs Raftis and Lucking gave advice on the preparation for and analysis of the Badimon study and flow cytometry. Dr Hunter performed the data and statistical analysis. Drs Hunter and Mills drafted the manuscript, and all the authors were involved in critical review. All authors read and approved the final manuscript.

## Sources of Funding

This work was supported by the British Heart Foundation (PG 11/27/24482; RG/10/9/28286) and the Colt Foundation. Dr Mills is supported by the British Heart Foundation Butler Senior Clinical Research Fellowship (FS/16/14/32023). Dr Newby is supported by the British Heart Foundation (CH/09/002) and is the recipient of a Wellcome Trust Senior Investigator Award (WT103782AIA).

## Clinical Perspective

### What Is New?

- Cardiovascular events are the leading cause of death among firefighters and are responsible for ≈45% of on-duty fatalities each year in the United States.
- The risk is substantially increased during fire suppression duties, and is likely to reflect factors including physical exertion, heat, and exposure to air pollution.
- In healthy firefighters, we assessed the effects of simulated fire suppression on thrombus formation, platelet activation, and forearm blood flow.
- Exposure to heat and physical exertion during fire suppression activates platelets, increases thrombus formation, and impairs vascular vasomotor function in firefighters: all key mechanisms in the pathogenesis of acute myocardial infarction.

### What Are the Clinical Implications?

- Increased thrombogenicity and impaired vascular function may be secondary to an increase in core body temperature and dehydration during fire suppression.
- Limiting the duration of exposure, active cooling, and effective rehydration are simple and inexpensive ways that might mitigate the risk posed by fire suppression.
- These findings suggest pathogenic mechanisms to explain the association between fire suppression activity and acute myocardial infarction in susceptible firefighters.
